# Critical appraisal of meta-analyses: an introductory guide for the practicing surgeon

**DOI:** 10.1186/1754-9493-3-16

**Published:** 2009-07-22

**Authors:** Nathan Lawrentschuk, Jonathan McCall, Ulrich Güller

**Affiliations:** 1 Department of Urology, University of Toronto, University Health Network, Toronto, Canada; 2 DCRI Communications Group, Duke Clinical Research Institute, Durham, NC, USA; 3 European Board Certified Colo-Proctology (EBSQ), Department of Surgery, Division of Visceral Surgery and Transplantation, Inselspital, University of Bern, CH-3010 Bern, Switzerland; 4 Department of Surgery, University of Toronto, Toronto, Canada

## Abstract

Meta-analyses are an essential tool of clinical research. Meta-analyses of individual randomized controlled trials frequently constitute the highest possible level of scientific evidence for a given research question and allow surgeons to rapidly gain a comprehensive understanding of an important clinical issue. Moreover, meta-analyses often serve as cornerstones for evidence-based surgery, treatment guidelines, and knowledge transfer. Given the importance of meta-analyses to the medical (and surgical) knowledge base, it is of cardinal importance that surgeons have a basic grasp of the principles that guide a high-quality meta-analysis, and be able to weigh objectively the advantages and potential pitfalls of this clinical research tool. Unfortunately, surgeons are often ill-prepared to successfully conduct, critically appraise, and correctly interpret meta-analyses. The objective of this educational review is to provide surgeons with a brief introductory overview of the knowledge and skills required for understanding and critically appraising surgical meta-analyses as well as assessing their implications for their own surgical practice.

## Background

The statistical tool of meta-analysis is used with increasing frequency in surgical research. A recent review demonstrates that over the past decade, appearances of meta-analyses in the medical literature have increased by fourfold [1]. The vast majority of meta-analyses combine results from different randomized controlled trials (RCTs) and, to a much lesser extent, cohort studies or case-control studies. In the interest of brevity, we will focus this short educational review on meta-analyses of RCTs only.

Since their invention and subsequent application in the medical literature in the early 20^th ^century [2], meta-analyses have continuously evolved. The practice of performing high-quality, methodologically sound, and critically evaluated meta-analyses culminated in the creation of the Cochrane Group. Named for Archie Cochrane, a British researcher who contributed greatly to the development of modern epidemiology, the Cochrane group was established 15 years ago and is an international collaboration of over 10,000 investigators who appraise and compile high-quality meta-analyses on numerous topics, with over 1,600 published to date [3].

Given the ubiquity of meta-analyses in the current flourishing culture of evidence-based medicine, it is imperative for the practicing surgeon to acquire a basic understanding of the advantages and limitations of meta-analyses. Unfortunately, many surgeons lack a solid foundation in this essential area of knowledge. The present article represents an invited review and is based on different educational articles by the senior author (U.G.) [4-9]. Our objective is to provide a brief introductory overview of the techniques used to perform a meta-analysis and discuss some of the advantages and potential shortcomings of this statistical tool.

### Basic Statistical Background

If we are to understand and successfully apply the tool of meta-analysis, we must first briefly review some important statistical concepts, which have been described in greater detail by the senior author [6-8]. In statistical terms, there are two basic ways study findings can err. First, the study results might lead to an erroneous conclusion that a statistically significant difference exists between study groups when in reality it *does not *(Table 1, cell B). The second form of error is the reverse of the first: the study results might lead to an erroneous conclusion that there is no significant difference between the study groups when in reality a difference *does *exist (Table 1, cell C).

**Table 1:** Type I (alpha) and type II (beta) error [6].

		**Truth in the overall patient population**	
		Treatment difference	No treatment difference

**Study results**	Treatment difference	A. Correct conclusion	B. *Type I error *(false-positive result)
	
	No treatment difference	C. *Type II error *(false-negative result)	D. Correct conclusion

The first situation represents false-positive result and is called a *type I error*. The bound that we put on the probability of committing a type I error is named *alpha*, also referred to as the *level of statistical significance *or *significance level*. The second situation represents a false-negative result and is called a *type II error *or *beta error*. *Beta*, the false-negative rate, is complementary to the *power *of a study [6], which is defined as the probability of finding a statistically significant result (i.e., rejecting the null hypothesis) in a study when a true difference exists between or among the groups of subjects being compared.

Often in biomedical research alpha is set at 0.05, meaning that a 5% chance of obtaining a false-positive result (i.e., the results show a statistically significant difference even though no real difference exists) is considered acceptable. Alpha is the benchmark to which *p *values are compared. If the *p *value is larger than alpha, a result is said to be non-significant. On the other hand, if the *p *value is smaller than the benchmark alpha, the findings are considered statistically significant.

Although it might at first seem reasonable to assume that both alpha and beta errors could be set at the same level of 5%, a false-positive finding is often considered potentially more harmful than a false-negative result (e.g., finding a surgical procedure to be beneficial to patients when no benefit actually exists). Thus, in medical science, beta is commonly set between 0.2 and 0.1. As the power of a study is complementary to the beta error, type II errors of 0.2, 0.15, and 0.1 correspond to statistical powers of 80% (1.0-0.2), 85% (1.0-0.15), and 90% (1-0.10), respectively.

It is important to recall that the sample size of a study is directly proportional to the power: the larger the sample size, the higher the power. This simple statistical concept is of tremendous importance to meta-analyses. A meta-analysis combines different RCTs to increase the overall sample size, thus increasing the statistical power. This increase in statistical power in turn shrinks the value of beta, with result that the chances of a false-negative finding are minimized in a well-performed meta-analysis.

Finally, effect size forms a critical part of evaluating meta-analyses, as has been described by the senior author in greater detail elsewhere [9]. In summary, a meta-analysis combines the results of multiple studies that test a similar research hypothesis. Thererfore, the findings of individual RCTs (effect sizes) are combined using statistical techniques into an overall effect size, sometimes called meta-effect size. The meta-effect size is a more powerful and accurate estimate of the true effect sized compared with individual single studies.

### Steps in Performing a Meta-Analysis

A thorough understanding and appreciation of all of the steps in the process of performing a meta-analysis is essential for the reader. This importance is reflected in an admonition contained within the Cochrane Handbook: eager clinical trialists seeking to skip steps on the way to performing the statistical calculations for a meta-analysis are greeted with the warning: "*Don't start here!*" [10]. The different steps [11,12] include:

1. **Formulate a research question**.

2. **State the *a priori *hypothesis **(a hypothesis generated *prior *to collecting the data). This step is vital to ensuring the validity of the meta-analysis. No matter how tempting it might be to form hypotheses as interesting correlations or patterns appear in the collected data, doing so is likely to bias the meta-analyses irretrievably and diminish its validity.

3. **Write a protocol **in which the research question, as well as inclusion and exclusion criteria for the trials to be pooled in the meta-analysis, are clearly described.

4. **Perform a thorough literature search **using several different search engines (e.g., PubMed, Embase, Cochrane, Google Scholar, etc.). Be sure to formally document the search strategies used and the results that the searches retrieved; the sensitivity and precision of the literature search is itself likely to affect the ultimate validity of the findings [13-16]. A non-electronic hand searching of the literature may also be useful, despite the time and effort required [17].

5. **Perform a quality assessment **(critical appraisal) and extraction of studies (usually performed by two *independent *investigators).

6. **Extract the data **from the RCTs.

7. **Perform a statistical analysis **(including a sensitivity analysis).

8. **State conclusions and provide recommendations**.

**Figure 1:**
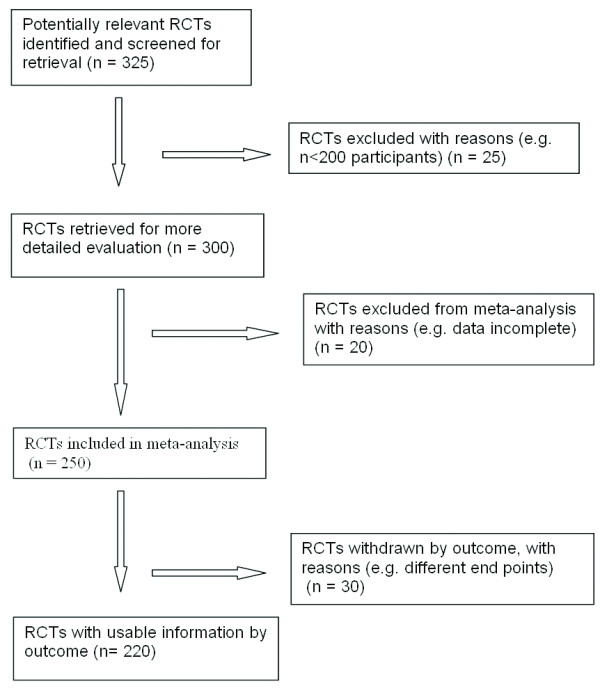
**Hypothetical flow chart based on the QUOROM statement**. This diagram represents a hypothetical flow chart based on the QUOROM statement flow diagram (modified from that provided by the Cochrane collaboration, ). The object of such diagrams is to improve the quality of reports of meta-analyses of randomized controlled trials (RCT).

Researchers may also wish to consult the guidelines established by the Quality of Reporting of Meta-Analyses (QUOROM) group [18]. The QUORUM guidelines, much like the CONSORT guidelines for reporting RCTs in the peer-reviewed literature [19], contain a listing of essential steps and items that must be included in a well-conducted meta-analysis. The QUOROM guidelines also incorporate flowcharts and checklists to be used when drafting a report on a meta-analytical study (Figure 1).

As we examine in detail the steps of performing a meta-analysis, it is important to emphasize particular aspects of the process. First, as with any study, the value of a meta-analysis can be assessed using the mnemonic '**FINER**'. That is, the study must be ***F***easible, ***I***nteresting, ***N***ovel, ***E***thical, and ***R***elevant [20]. If a meta-analysis is feasible and ethical but not relevant and novel, it will be worthless–there is little to be gained from answering questions without any clinical relevance, or re-hashing research questions that have been thoroughly and definitively addressed.

Second, the literature search for relevant RCTs should be undertaken only after a protocol has been written, with *a priori *hypotheses and inclusion and exclusion criteria clearly defined. Ideally, at least two independent investigators should search for studies that meet inclusion criteria. Further, the search should be as comprehensive as possible; that is, not limited only to Medline, but rather spread among other scientific databases (e.g., Embase, Cochrane, Google Scholar, etc.) to minimize the possibility of omitting a study of interest simply because of the vagaries of data collection or indexing. Similarly, limits on the language of the publication, date, country of origin, etc. should be avoided if possible. Moreover, as explained in greater detail below, performing a systematic search for unpublished studies is imperative.

Finally, two independent investigators must assess the quality and suitability of retrieved studies. Assessments and decisions regarding inclusion of a given study in the meta-analysis should be based on the inclusion and exclusion criteria outlined in the protocol.

### Interpretation of Forest Plots

**Figure 2:**
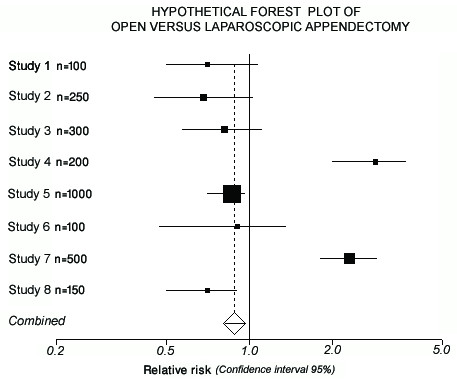
**A simplified, hypothetical example of a forest (meta-analysis) plot**. This figure represents a simplified, hypothetical example of a forest (meta-analysis) plot demonstrating eight RCTs comparing laparoscopic versus open appendectomy with respect to postoperative wound infections. Each RCT is represented by a square (the odds ratio found for this trial) and a horizontal line, which represents the 95% confidence interval. If the square is to the left of the vertical line of no effect (odds ratio = 1, e.g. studies 1, 2, 3, 5, 6, and 8), the study favors laparoscopic appendectomy; if the square is to the right of the line (e. g. studies 4 and 7), then open appendectomy is favored. If the 95% confidence interval crosses the line of no difference (odds ratio = 1), then the trial is not statistically significant (e.g., studies 1, 2, 3, and 6). Conversely, if the 95% confidence interval does not cross the line of no effect (odds ratio = 1), then this trial yields a statistically significant difference. Studies 4 and 7 found a significant advantage in favor of open appendectomy, whereas studies 5 and 8 found significantly less wound infection in the group randomized to laparoscopic appendectomy, indicating considerable heterogeneity. The size of the squares varies with respect to the sample size of each individual trial: the larger the sample size, the larger the square will be. An overall (pooled) effect is represented by the diamond. In this case, the overall results demonstrate a statistically significant reduction of postoperative wound infections in the group randomized to laparoscopic appendectomy (Forest plot created with StatsDirect v. 2.7.2; StatsDirect Ltd., Cheshire, UK).

In a meta-analysis, the combined data from the various selected RCTs are typically presented as forest plots (Figure 2) [21]. Correct interpretation of these forest plots is crucial for the surgical reader and deserves some discussion. Forest plots use boxes and "whiskers" (horizontal lines indicating the spread of the 95% confidence interval) to represent individual trials. On close inspection, the reader will note that the size of the boxes varies among the different studies represented. In fact, box size correlates directly with the sample size (number of patients enrolled) of an RCT. For instance, in Figure 2, Study 5 has a larger sample size (larger box) compared with Studies 7 and 8. Such larger trials will carry more weight in the meta-analysis than smaller trials.

As mentioned above, the whiskers represent the 95% confidence intervals. These 95% confidence intervals comprise the range of values for which you can be 95% confident that the true value is included, and help provide the reader with an appreciation of the reliability of the results. The wider the 95% confidence intervals, the higher the uncertainty that the reported results are accurate [7]. The width of the 95% confidence intervals is indirectly proportional to the sample size of the RCT: if the trial includes a large number of patients (e.g., Study 5, Figure 2), the width of the 95% confidence intervals will be narrow; as sample sizes dwindle, the 95% confidence intervals correspondingly grows wider.

In addition to providing information on the reliability of the results, the whiskers of a 95% confidence interval can inform the reader as to whether the study was statistically significant [7]. If the confidence interval crosses the vertical line of no effect (0 for a difference between two groups and 1 for a ratio of two groups), then that trial result, taken individually, is *not *statistically significant (e.g., Study 6 in Figure 2). Conversely, if the confidence interval does not cross the vertical line of no effect, the result *is *statistically significant (e.g., Studies 5 and 8 in Figure 2). The overall result (summary effect) is represented by the diamond shape. In Figure 2, the confidence interval does not cross the line of no effect and thus represents a statistically significant overall result (*p *< 0.05).

### Advantages of Meta-Analysis

When performed properly, meta-analyses have a number of important advantages over individual RCTs. These include:

#### Increasing power

The primary advantage of meta-analyses is an increase in statistical power over that of individual RCTs. As previously mentioned, power is defined as the probability of detecting a statistically significant result if the patient samples are truly different [7,9]. For various reasons that are often difficult to predict beforehand, RCTs frequently prove to be underpowered. In other words, they enroll too few patients to prove that a detected difference, even when clinically relevant, is statistically significant [7,22]. The result is a negative study; in other words, the *p *value exceeds the threshold for significance. In these small RCTs it may be unclear whether the lack of statistical significance truly reflects the fact that there is no difference between treatments, or whether the sample size was simply too small to demonstrate that a detected difference was significant. As mentioned earlier, meta-analyses may overcome this limitation by combining different RCTs, thereby increasing overall sample size (and with it, statistical power) in a way that would simply not be feasible if one were to attempt creating a single RCT with a comparable sample size.

#### Providing a unifying conclusion

Often, in a given area of interest, various RCTs may provide contradictory results. Quite commonly, this occurs for the same reason discussed above: the sample sizes for the various RCTs were not sufficient to ensure a definitive answer to the research question. This confusion, however, can be abated by applying the tool of meta-analysis, which can reveal an underlying unifying conclusion among seemingly contradictory study findings.

For a practical example, let us consider the role of neoadjuvant chemotherapy prior to radical cystectomy with extended lymphadenectomy in the treatment of bladder cancer. There have been many conflicting RCTs performed in this clinical arena, some of which found a statistically significant overall survival advantage with neoadjuvant chemotherapy in addition to radical cystectomy, while others found no such advantage. In order to elucidate this issue, a meta-analysis combining the various RCTs was performed. The meta-analysis demonstrated a 5% absolute improvement in overall survival at 5 years when more than 3000 patients from 11 RCTs were analysed [22]. In this case, a meta-analysis helped reveal a unifying conclusion and led to important gains in knowledge as well as direct benefit for patients.

### Limitations of Meta-Analyses

When conducted with appropriate statistical techniques and with high-quality data, findings from meta-analyses are considered to be the highest level of evidence (level 1a evidence) [23]. However, as the saying goes, "the devil is in the details." Despite their widespread acceptance, some authorities have remained skeptical about the overall usefulness and reliability of meta-analyses; moreover, the techniques used in a meta-analysis are quite sensitive to the care and skill of the persons performing the analysis. For this reason, the surgical reader should bear in mind a number of important caveats (summarized in Table 2).

**Table 2:** Checklist for the surgeon to critically appraise a meta-analysis

Has a research question/hypothesis been formulated *a priori *(before starting the meta-analysis)?
Is the research question FINER[20] (***F***easible, ***I***nteresting, ***N***ovel, ***E***thical, and ***R***elevant)?

Is the meta-analysis based on a written protocol that clearly outlines research question, primary and secondary outcomes, and inclusion and exclusion criteria?

Has a thorough literature search been performed? Have different search engines (PubMed, Embase, Cochrane library, etc.) been used to identify relevant literature?

Did the authors look for unpublished data, for negative studies, and for publications in non-English languages to minimize retrieval, language, and publication bias?

Was a strategy to exclude individual studies clearly outlined in the publication?

Did two investigators independently perform the quality assessment of the individual studies?

Were sensitivity analyses performed?

#### Garbage in-garbage out phenomenon

It is quite possible for a researcher to apply methodologically sound meta-analytical techniques to suboptimal data. Unfortunately no amount of statistical technique can improve the fundamental quality of the data being combined for the meta-analysis. If the individual RCTs that make up the meta-analysis are themselves poorly designed and poorly conducted, the meta-analysis summarizing these trials will be of correspondingly limited reliability. Remember: garbage in-garbage out [8]!

It is important to acknowledge that RCTs in surgery are themselves subject to their own particular challenges and sets of biases as has been discussed previously by the senior author in greater detail elsewhere [4,5]. Briefly, typical caveats of surgical trials include limitations such as low external validity (poor generalizability), difficulty of blinding patients and investigators, co-intervention bias, lost-to-follow-up bias, and performance bias. Because it is often difficult to control for these biases it is therefore important that the astute reader of a meta-analysis evaluate the individual RCTs to assess the overall quality of the meta-analysis [4,9].

#### Publication bias

There is a well-known phenomenon, extensively documented in the published literature, whereby positive trials (studies that produce a statistically significant result) are much more likely to be published than so-called negative trials (studies producing no statistically significant association), or trials that produce equivocal results [1,24]. There are a number of possible reasons for the existence of such a bias, but although editorial bias in medical journals is often held as a culprit, there is some evidence to suggest that the bias may also arise when investigators or sponsors simply decide not to write and publish negative results[25]. Regardless of its origin, however, publication bias may lead to overestimating the effect of the intervention being examined in the meta-analysis. Thus, in order to ensure the reliability of a meta-analysis, one must systematically search for negative trials for inclusion.

A variety of methods are used to detect potential publication biases, including graphical depiction through the use of funnel plots [1,26]. In summary, such plots are scatter diagrams of the estimated treatment effects in the individual studies against the study size. Small studies will give more variable estimates and hence greater scatter. When completed, the plot should have a symmetrical appearance like that of a triangle or inverted funnel. An asymmetry in the funnel plot may reflect the possibility that smaller studies were not published due to non-significant findings, thus indicating publication bias. Funnel plots are easy-to-use, practical tools and should be employed systematically to detect and possibly prevent publication bias [27].

Recent consensus statements by the World Health Organization (WHO) and the International Council of Medical Journal Editors (ICMJE) [28] have led to requirements that any RCT be registered with http://www.clinicaltrials.gov/  before commencing patient accrual; failing to do so may render the study unable to be published by a large and growing number of peer-reviewed journals and additional sanctions may apply to researchers who neglect to register clinical trials. Although even the most finely-honed searches may fail to reveal negative data that has simply been "shelved" by investigators or sponsors, any author attempting a meta-analysis should exhaust every possible avenue for obtaining the most complete set of data possible.

Retrieval bias and language bias are often considered as two facets of publication bias and will be discussed below.

##### Retrieval bias

This bias refers to a potential distortion of the findings of a meta-analysis due to the overlooking or exclusion of relevant studies that merit inclusion in the meta-analysis. Retrieval bias may be the result of suboptimal search of electronic databases and failing to identify important unpublished results. It is critically important to search a variety of different databases [29]. Novice researchers should be particularly attentive to carefully choosing search terms. The appropriate use of Medical Search Headers (MeSH) keywords and Boolean search strings can help maximize the retrieval of relevant articles; in particular, relatively inexperienced researchers may wish to experiment with search tutorials, such as those offered by PubMed, and refer to the growing body of literature regarding the optimization of literature searches.

##### Language bias

Language bias is closely related with retrieval bias. It refers to a potential distortion of the results of a meta-analysis due to a failure to identify relevant study findings published in languages other than English. If a methodologically sound meta-analysis is performed, the investigators must systematically search for relevant studies outside of the scientific English literature [30].

#### Heterogeneity

Because perioperative care and surgical techniques are not necessarily uniform or easily standardized [9] meta-analyses are consequently more difficult to perform on surgical interventions than in drug trials. Dealing with such heterogeneity may be challenging: Not only is there a risk of heterogeneity *within *the RCT, but this heterogeneity may be amplified when combining different trials. Therefore, if the heterogeneity among the various RCTs included in a meta-analysis is high, the investigator risks comparing apples and oranges. Although it may occur by chance alone, heterogeneity in surgical trials is most often associated with variation in technical ability among surgeons [9].

Well-designed and rigorously performed multi-center surgical RCTs now typically contain some validation of standardized surgical technique. For instance, standardization can be achieved through peer review of surgical procedures. A good example of this is the COST trial, which compared open and laparoscopic colectomies for cancer [31]. Before the laparoscopic surgeons were allowed to enroll patients into the trial, their surgical techniques were submitted to peer review, thereby ensuring a baseline standard. The peer review process diminishes the risk of suboptimal technical skills acting as a potential confounder and enhances homogeneity of surgical skills among similar trials.

The extent of heterogeneity among various trials can be determined through the application of the method of sensitivity analysis. In this case, what is important is not whether the subgroups remain statistically significant with respect to the overall research question, but whether any statistically significant differences exist among them. Also, if homogeneity exists, then omitting a certain trial from the analysis should not change the overall result. Conversely, if heterogeneity is present, then omitting a key trial may well change the pooled estimate. It follows that the more similar the included RCTs are, the less the degree of heterogeneity. If too much heterogeneity is found among different RCTs in the area of interest, performing a meta-analysis may not be feasible, and the reporting of different studies in a systematic review may be more appropriate.

## Conclusion

Basic knowledge of the advantages and limitations of meta-analyses is essential to the practicing surgeon. Although meta-analyses are considered the highest level of evidence and an essential part of medical research, the clinician must be aware of their potential limitations, either when conducting a meta-analysis or interpreting the findings from one. Combining independent RCTs using statistical techniques will increase the statistical power in the context of the research question, but this will not necessarily translate into a higher-confidence conclusion if the individual studies that make up the meta-analysis are not themselves sufficiently well-designed and conducted.

## Competing interests

The authors declare that they have no competing interests. JM is an employee of the Duke Clinical Research Institute but was not compensated in any fashion for his work on this manuscript.

## Authors' contributions

NL carried out conception and design of the review and drafted the manuscript. JM participated in its design and coordination and helped to draft the manuscript. UG conceived of the study, and participated in its design, coordination and supervision. All authors read and approved the final manuscript.

## References

[B1] Mahid SS, Hornung CA, Minor KS, Turina M, Galandiuk S (2006). Systematic reviews and meta-analysis for the surgeon scientist. Br J Surg.

[B2] L'Abbe KA, Detsky AS, O'Rourke K (1987). Meta-analysis in clinical research. Ann Intern Med.

[B3] Chalmers I (1993). The Cochrane collaboration: preparing, maintaining, and disseminating systematic reviews of the effects of health care. Ann N Y Acad Sci.

[B4] Ridgeway P, Guller U (2009). Interpreting study designs in surgical research – a practical guide for surgeons and surgical residents. Journal of the American College of Surgeons.

[B5] Herrle F, Guller U (2009). Evidence-Based Surgery Series: Part 2: Interpreting Meta-Analyses and Systematic Reviews in Surgical Research – A Practical Guide for Surgeons and Surgical Residents. Zeitschrift fuer Herz- Thorax- und Gefaesschirurgie.

[B6] Guller U, Oertli D (2005). Sample size matters: a guide for surgeons. World J Surg.

[B7] Guller U, DeLong ER (2004). Interpreting statistics in medical literature: a vade mecum for surgeons. J Am Coll Surg.

[B8] Guller U (2008). Caveats in the interpretation of the surgical literature. Br J Surg.

[B9] Guller U (2006). Surgical outcomes research based on administrative data: inferior or complementary to prospective randomized clinical trials?. World J Surg.

[B10] Deeks JJ, Macaskill P, Irwig L (2005). The performance of tests of publication bias and other sample size effects in systematic reviews of diagnostic test accuracy was assessed. J Clin Epidemiol.

[B11] Sauerland S, Seiler CM (2005). Role of systematic reviews and meta-analysis in evidence-based medicine. World J Surg.

[B12] Neugebauer E, Lefering R, McPeek B, Wood-Dauphinee S, Troidi H, McKneally M, Mulder D (1998). Systematically reviewing previous work. Surgical Research: Basic Principles and Clinical Practice.

[B13] Wilczynski NL, Haynes RB (2004). Developing optimal search strategies for detecting clinically sound prognostic studies in MEDLINE: an analytic survey. BMC Med.

[B14] Robinson KA, Dickersin K (2002). Development of a highly sensitive search strategy for the retrieval of reports of controlled trials using PubMed. Int J Epidemiol.

[B15] Glanville JM, Lefebvre C, Miles JN, Camosso-Stefinovic J (2006). How to identify randomized controlled trials in MEDLINE: ten years on. J Med Libr Assoc.

[B16] Dickersin K, Scherer R, Lefebvre C (1994). Identifying relevant studies for systematic reviews. BMJ.

[B17] Hopewell S, Clarke M, Lefebvre C, Scherer R (2007). Handsearching versus electronic searching to identify reports of randomized trials. Cochrane Database Syst Rev.

[B18] Moher D, Cook DJ, Eastwood S, Olkin I, Rennie D, Stroup DF (1999). Improving the quality of reports of meta-analyses of randomised controlled trials: the QUOROM statement. Quality of Reporting of Meta-analyses. Lancet.

[B19] CONSORT Statement. CONSORT Transparent Reporting of Trials.

[B20] Hulley S, Cummings S, Browner W, Grady D, Newman T (2001). Designing clinical research: an epidemiologic approach.

[B21] Lewis S, Clarke M (2001). Forest plots: trying to see the wood and the trees. BMJ.

[B22] Guller U (2008). Meta-Analyses: Advantages and Caveats. Swiss Knife.

[B23] Phillips B, Ball C, Badenoch D, Straus S, Haynes B, Dawes M (2008). Oxford Centre for Evidence-based Medicine Levels of Evidence (May 2001). BJU International.

[B24] Dickersin K (1990). The existence of publication bias and risk factors for its occurrence. JAMA.

[B25] Olson CM, Rennie D, Cook D, Dickersin K, Flanagin A, Hogan JW, Zhu Q, Reiling J, Pace B (2002). Publication bias in editorial decision making. JAMA.

[B26] Egger M, Smith GD (1995). Misleading meta-analysis. BMJ.

[B27] Mayer EK, Bottle A, Rao C, Darzi AW, Athanasiou T (2009). Funnel plots and their emerging application in surgery. Ann Surg.

[B28] Deangelis CD, Drazen JM, Frizelle FA, Haug C, Hoey J, Horton R, Kotzin S, Laine C, Marusic A, Overbeke AJ, Schroeder TV, Sox HC, Weyden MB Van Der (2005). Is this clinical trial fully registered? A statement from the International Committee of Medical Journal Editors. JAMA.

[B29] Greenhalgh T (1997). Papers that summarise other papers (systematic reviews and meta-analyses). BMJ.

[B30] Parnaby CN, MacDonald AJ, Jenkins JT (2009). Sham feed or sham? A meta-analysis of randomized clinical trials assessing the effect of gum chewing on gut function after elective colorectal surgery. Int J Colorectal Dis.

[B31] (2004). A comparison of laparoscopically assisted and open colectomy for colon cancer. N Engl J Med.

